# Hospital Mergers, Hospital Choice, and Care Quality for Pregnant Enrollees in Medicaid

**DOI:** 10.1001/jamahealthforum.2025.5334

**Published:** 2025-12-05

**Authors:** Sunita M. Desai, Prianca Padmanabhan, Sherry Glied, Eric T. Roberts

**Affiliations:** 1 Department of Population Health, NYU Grossman School of Medicine, New York, New York; 2 NYU Wagner School of Public Service, New York, New York; 3 University of Pennsylvania, Philadelphia

## Abstract

**Importance:**

Despite increasing consolidation in the US hospital market, little is known about how these mergers influence labor and delivery admissions and obstetric outcomes for patients with Medicaid.

**Objective:**

To assess whether hospital mergers are associated with changes in patient flows to hospitals and care quality for pregnant Medicaid enrollees.

**Design, Setting, and Participants:**

This cross-sectional study used a stacked difference-in-differences design to examine mergers occurring between 2004 and 2011. Changes in outcomes were examined in the 3 years before vs 3 years after a merger. Estimates were compared by urban vs rural settings and for Medicaid patients relative to privately insured patients. Labor and delivery admissions in 9 US states accounted for approximately 25% of all Medicaid-covered births nationwide during the study period. Medicaid-enrolled patients admitted for labor and delivery residing in counties that experienced a single hospital merger (intervention) or matched comparison counties without a merger during the same period were included. The analysis took place between December 2023 and April 2025.

**Exposure:**

Residing in a county that experienced a hospital merger.

**Outcomes:**

Outcomes included patient travel distance for delivery, admissions to safety net hospitals, and admissions to hospitals with a neonatal intensive care unit (NICU), indicating advanced obstetric capabilities. Obstetric quality outcomes included obstetric trauma for instrument-assisted and non–instrument-assisted admissions and in-hospital mortality.

**Results:**

The analysis included 527 499 Medicaid labor and delivery admissions across 30 merger and 28 nonmerger counties. The mean (SD) age across Medicaid labor and delivery admissions was 25.8 (5.9) years, and all were female individuals. In addition, 15.4% were Black, 19.9% were Hispanic, and 20.5% were White individuals. County-level exposure to a merger was associated with an adjusted 0.5-mile (95% CI, 0.1-1.0) increase in travel distance (an 8% increase from the 6.3-mile premerger baseline), a 9.2–percentage point (95% CI, 2.3-16.1) increase in admissions to safety net hospitals, and a 7.9–percentage point (95% CI, –11.2 to –4.6) decrease in admissions to NICU-equipped hospitals. One obstetric trauma measure (among non–instrument-assisted deliveries) increased slightly (0.4 percentage points; 95% CI, 0.2-0.6), whereas in-hospital mortality was unchanged. Urban counties experienced a decrease in admissions to NICU-equipped hospitals, whereas rural counties experienced increases. Compared with privately insured patients, Medicaid enrollees had larger increases in travel distance (0.6; 95% CI, 0.0-1.1) and safety net admissions (6.8; 95% CI, 1-12.6), but similar changes in NICU-equipped hospital admissions (−0.1; 95% CI, −2.4 to 2.3).

**Conclusions and Relevance:**

This cross-sectional study of labor and delivery admissions among patients with Medicaid found that hospital mergers were associated with an increased probability of admission to safety-net hospitals, lower probability of NICU-equipped hospitals, and worsening in 1 obstetric quality metric. These changes differed for Medicaid vs commercially insured patients and varied among Medicaid patients in urban vs rural markets. The findings underscore the importance of considering local market structure and potential adverse impacts on low-income populations when evaluating proposed hospital mergers.

## Key Points

**Question:**

Are hospital mergers associated with changes in the hospitals where Medicaid enrollees are admitted for labor and delivery and with obstetric outcomes?

**Findings:**

In this difference-in-differences study of labor and delivery admissions among 527 499 Medicaid enrollees, county-level exposure to a hospital merger was associated with increased travel distances, shifts in admissions toward safety-net hospitals and away from hospitals with neonatal intensive care units, and a small worsening in 1 quality metric. Other obstetric outcomes did not change significantly; associations varied across urban and rural areas.

**Meaning:**

Hospital mergers may exacerbate access barriers for Medicaid patients, underscoring the need to consider local market structures when developing policies for safety net populations and evaluating mergers.

## Teaser

This study uses hospital discharge data for labor and delivery admissions across 9 states and over 14 years to describe associations between hospital mergers and access to and quality of care for Medicaid patients admitted for childbirth.

## Introduction

Hospital mergers have led to more concentrated hospital markets, often dominated by a small number of large hospital systems.^1,2^ Prior research documented that such mergers lead to higher prices paid by commercial insurers without corresponding improvements in quality.^3,4,5,6,7,8,9,10^ Despite the frequency of mergers and concerns about their effects, there is limited evidence on how mergers impact low-income populations, particularly Medicaid enrollees.^11,12^

Economic theory and prior research offer competing predictions about the consequences of hospital mergers for Medicaid enrollees. Mergers could reduce access to and quality of care because hospitals may restructure service lines to prioritize newly more profitable commercially insured patients. Alternatively, mergers that improve operational efficiencies and financial performance could enable hospitals to sustain Medicaid-dominant services, such as obstetrical care.^13^ The effects of mergers may also vary by market characteristics. For example, in rural areas, which often have constrained financial resources and limited hospital access, mergers that consolidate hospital services could exacerbate existing access challenges.

One area of particular importance for Medicaid is the impact of hospital mergers on childbirth admissions. Medicaid covers approximately 40% of births in the US.^14^ Although prior literature has examined the consequences of hospital and maternity ward closures,^15,16,17,18^ the effects of mergers on maternity services may be different and depend on whether services are eliminated or centralized under mergers, how referral patterns shift, and how patients access care. Research examining New York state found that as hospital market concentration increased due to mergers, Medicaid labor and delivery admissions shifted from nonprofit to public hospitals, which are frequently resource constrained and may therefore provide lower-quality care. However, that analysis was limited to a single state, did not separately examine rural markets, and did not explore other hospital characteristics or quality outcomes.

This study used hospital discharge data for labor and delivery admissions across 9 states and over 14 years to describe associations between hospital mergers and access to and quality of care for Medicaid patients admitted for childbirth. In addition, we investigated whether changes associated with mergers differed between Medicaid-enrolled patients in urban and rural markets. We also analyzed whether changes associated with mergers reflected broader shifts affecting all patients or disproportionately impacted Medicaid enrollees compared with privately insured individuals.

## Methods

This study is reported in accordance with the Strengthening the Reporting of Observational Studies in Epidemiology (STROBE) reporting guidelines, and the protocol was reviewed and approved by the NYU Langone Health institutional review board. The NYU Langone Health Institutional Review Board approved the study and waived the requirement for informed consent because data were deidentified. The analysis took place between December 2023 and April 2025. Our empirical strategy was a stacked difference-in-differences design that accounted for variation in the timing of mergers.^19,20,21,22^ We used an intention-to-treat approach that compared changes in outcomes among patients who had labor and delivery admissions and resided in counties that experienced a hospital merger to contemporaneous changes among patients in similar counties without a hospital merger.

### Data Sources

This study used data from 3 main sources. First, we used Healthcare Cost Utilization Project State Inpatient Data (HCUP SID) for 9 states (Arizona, Colorado, Florida, New Jersey, New York, North Carolina, Utah, Vermont, and Washington), which together accounted for approximately 25% of births nationally.^23^ The HCUP SID contained discharge records for all inpatient hospital admissions in a state including diagnoses, outcomes, payer type (eg, Medicaid, private, Medicare), the hospital, patient zip code, and certain demographic characteristics (eg, race and ethnicity). Second, we used American Hospital Association (AHA) Annual Survey for information on hospital characteristics and location. Third, we used comprehensive national data on US hospital mergers between 2004 and 2011 from Cooper et al.^1^ These data defined mergers based on transactions that resulted in ownership changes but did not capture looser affiliations or partnerships.

### Study Population

Our analytic sample comprised inpatient Medicaid admissions for labor and delivery (admissions having major diagnostic category 14) in a balanced panel of merger (intervention) and matched nonmerger (comparison) counties. Our primary analyses focused on patients whose primary insurance at the time of admission was Medicaid. To contrast the effects of mergers on publicly vs privately insured patients, in secondary analyses, we also included patients with private insurance.

Merger counties were defined as counties with exactly 1 hospital that was acquired between the years 2004 and 2011, allowing for 3 years of premerger and postmerger data. We dropped the year of the merger as a transition year and excluded counties that experienced multiple mergers during the study period.

We used propensity score matching to select comparison counties with similar characteristics as merger counties. This approach balanced the intervention and control population on baseline county characteristics (number of hospitals, total number of hospital beds, baseline Herfindahl-Hirschman Index, and county-level uninsured rate) and premerger trends in the Herfindahl-Hirschman Index, the proportion of admissions to a safety net hospital, and the proportion of admissions to a hospital with a neonatal intensive care unit (NICU) (eTables 1 and 2 in Supplement 1).

### Outcomes

We constructed several outcomes to examine whether mergers were associated with changes in the hospitals where patients go to give birth. First, we examined the distance a patient traveled to a hospital for labor and delivery. We measured this as the straight-line distance from the geographic centroid of the patient’s zip code of residence to the hospital.

Second, we examined the probability that a patient gave birth at a safety net hospital. A hospital’s safety-net status was assessed based on the ratio of its Medicaid and uninsured patient mix to that of all hospital admissions for county residents at baseline, defined as 3 years prior to the merger; a higher ratio indicates that the hospital serves a proportionately larger share of Medicaid and uninsured patients compared with the patient mix in the county. Hospitals with ratios in the top quartile were classified as safety net.

Third, we examined whether patients gave birth at hospitals with an NICU. This outcome serves as a labor- and delivery-specific measure of hospital resources and capacity because hospitals with NICUs tend to have greater clinical capabilities and resources for managing high-risk deliveries and neonatal complications.^18,24^ These NICU and safety net hospital outcomes capture both changes in hospital availability and shifts in where patients deliver, reflecting a combination of supply-side changes and patient choices. In a secondary analysis, to isolate changes in the supply of NICUs, we examined whether there was an NICU in the patient’s market.

We also assessed the effect of hospital mergers on Agency for Healthcare Research and Quality (AHRQ) Patient Safety Indicators related to obstetric outcomes, including the probability of obstetric trauma, which we analyzed separately for vaginal deliveries with and without the use of instruments (eg, forceps or vacuum extraction).^25^ Obstetric trauma is a key indicator of maternal safety and quality of obstetric care. We also examined in-hospital maternal mortality, a rare but critical outcome that serves as a direct measure of severe maternal complications and overall quality of care during childbirth.

### Statistical Analysis

We used a stacked difference-in-differences design that compared premerger vs postmerger changes in outcomes among patients in counties experiencing a hospital merger to changes among patients in similar counties without a merger during the study period.^19,26,27^ Each set of intervention and comparison counties was analyzed as a stack based on the merger year, and we compared changes over the same periods within each stack to account for secular trends. Our models aggregate treatment effects across all stacks to produce an overall estimate of outcome changes associated with mergers. We used a stacked rather than a standard difference-in-differences or 2-way fixed-effects design to avoid bias resulting from staggered treatment timing.

For each outcome, we estimated a linear regression model, where the unit of analysis was the labor and delivery admission and each admission was attributed to the county where the patient resided. Each model included an interaction between a postmerger year indicator and an indicator for whether the admitted patient resided in a merger (intervention) county, stack fixed effects (indicators for each set of intervention and matched comparison counties based on merger year), interactions between year and stack fixed effects, and interactions between county and stack fixed effects. Models also included covariates for patient age, race and ethnicity, and the Elixhauser Comorbidity Index.^28^ We estimated robust standard errors clustered by patient county of residence.^29,30^ Analyses were conducted using R statistical software (version 4.3.2, R Foundation).

The identifying assumption underlying our approach is that, in the absence of a merger, changes in outcomes in counties that experienced a merger would have been similar to those in counties that did not experience one. To assess this assumption, we conducted an event-study analysis to test for differences in premerger outcome trends between merger vs nonmerger counties (eFigure 1 in Supplement 1).

We conducted 2 further analyses. First, we stratified analyses by urban and rural location. Counties designated as metropolitan were defined as urban, and counties outside core-based statistical areas were defined as rural. Second, we added admissions for privately insured patients to the data and tested whether changes associated with mergers differed for Medicaid vs privately insured patients by fitting regression models that included interactions between patient insurance status with treatment group and postmerger indicators, stack fixed effects, stack-year fixed effects, and stack-county fixed effects, in addition to the terms in the main model.

## Results

### Descriptive Characteristics of Medicaid Labor and Delivery Admissions

We analyzed 318 587 Medicaid labor and delivery admissions in 30 merger counties and 208 912 admissions in 28 matched comparison counties (Table 1). The mean (SD) age across Medicaid labor and delivery admissions was 25.8 (5.9) years, and all were female individuals. In addition, 15.4% were Black, 19.9% were Hispanic, and 20.5% were White individuals. Patients in merger and nonmerger counties were similar along most dimensions, although there were differences in race and ethnicity composition between merger and nonmerger counties, with nonmerger counties including proportionately more admissions for Hispanic and White patients. In the premerger period, 90.5% of intervention admissions were in urban counties compared with 92.3% of admissions in comparison counties. In merger counties, the mean Hirschmann-Herfindahl Index increased from 3264 (1385) to 3638 (1311) from before to after the merger; in counties without a merger it and increased from 3399 (1326) to 3498 (1315) during concurrent periods.

**Table 1.** Descriptive Characteristics of Medicaid Labor and Delivery Admissions in Counties With Hospital Mergers and Matched Comparison Counties^a^

Characteristic	No. (%)			
	Counties with a hospital merger		Matched counties with no hospital merger in concurrent period	
	Premerger	Postmerger	Premerger	Postmerger
Total unique counties, No.	30	30	28	28
Total admissions, No.	159 580	159 007	101 734	107 178
Age, mean (SD), y	25.4 (5.9)	26.1 (5.9)	25.7 (6.0)	26.3 (5.9)
Race and ethnicity				
Black	24 629 (15.4)	22 984 (14.5)	17 938 (17.6)	19 074 (17.8)
Hispanic	31 713 (19.9)	52 178 (32.8)	29 038 (28.5)	28 229 (26.3)
White	32 739 (20.5)	48 752 (30.7)	33 948 (33.4)	38 110 (35.6)
Other^b^	70 499 (44.2)	35 093 (22.1)	20 810 (20.5)	21 765 (20.3)
Elixhauser Comorbidity Index				
0	137 713 (86.3)	130 481 (82.1)	87 102 (85.6)	86 297 (80.5)
1	18 396 (11.5)	23 466 (14.8)	12 097 (11.9)	16 748 (15.6)
2	2873 (1.8)	4242 (2.7)	2118 (2.1)	3450 (3.2)
≥3	598 (0.4)	818 (0.4)	417 (0.4)	683 (0.7)
HHI, mean (SD)^c^	3264 (1385)	3638 (1311)	3399 (1326)	3498 (1315)
CBSA category				
Metropolitan (urban)	144 465 (90.5)	142 467 (89.6)	93 855 (92.3)	99 591 (92.9)
Micropolitan	11 658 (7.3)	13 762 (8.7)	6696 (6.6)	6435 (6.0)
Outside CBSA (rural)	3457 (2.2)	2778 (1.8)	1183 (1.2)	1152 (1.1)

Abbreviations: CBSA, core-based statistical area; HHI, Herfindahl-Hirschman Index.

^a^ Premerger refers to the 3 years prior to the merger year and postmerger refers to the 3 years following the merger year.

^b^ Other included Asian or Pacific Islander, Native American, other, and missing.

^c^ The HHI measures hospital market concentration in a hospital’s service regions and is scaled from 0 to 10 000. Higher values indicate more concentrated markets. See Supplement 1 for details on the HHI measure.

Compared with our analytic sample, Medicaid patients in the 30 counties excluded for having multiple mergers were more likely to be Black and reside in urban areas (eTable 3 in Supplement 1). Descriptive characteristics for privately insured patients included in secondary analyses are in eTable 4 in Supplement 1.

### Association of Hospital Mergers With Medicaid Admission Outcomes

Among Medicaid admissions, the mean distance traveled by patients to the hospital during premerger years was 6.3 miles in merger counties (Table 2). Mergers were associated with an adjusted increase in distance traveled of 0.5 (95% CI, 0.1-1.0) miles.

**Table 2.** Stacked Difference-in-Differences Estimates of the Association Between County-Level Exposure to a Hospital Merger and Hospital Choice and Outcomes for 527 499 Medicaid Labor and Delivery Hospital Admissions^a^

Outcome	Premerger outcomes in counties with a hospital merger, %	Stacked difference-in-differences, estimate (95% CI)	
		Unadjusted	Adjusted
Patient access and hospital choice			
Patient distance to hospital, miles	6.3	0.5 (0.1 to 1)	0.5 (0.1 to 1)
Admission to safety net hospital	37.6	5.8 (−1.1 to 12.7)	9.2 (2.3 to 16.1)
Admission to hospital with a neonatal intensive care unit	72.2	−8.5 (−11.8 to −5.2)	−7.9 (−11.2 to −4.6)
Obstetric quality outcomes			
Obstetric trauma, instrument-assisted deliveries	13.7	1.2 (−1.2 to 3.6)	1.6 (−0.8 to 4)
Obstetric trauma, non–instrument-assisted deliveries	1.9	0.3 (0.1 to 0.5)	0.4 (0.2 to 0.6)
In-hospital death	0.01	0 (0 to 0)	0 (0 to 0)

^a^ Stacked difference-in-differences coefficient estimates and 95% CIs from the main analysis are presented as well as mean outcomes across admissions in merger counties during premerger years. The estimates represent the differential change in the outcome associated with a hospital merger occurring in the patient’s county compared with the change in the outcome for admissions in matched comparison counties not experiencing a merger during the concurrent time period. The premerger period includes the 3 years prior to the merger, and the postmerger period includes the 3 years following the merger year. The merger year is excluded as a transition year. Each model controls for an interaction between treatment and postmerger indicators, stack fixed effects, interactions between year and stack fixed effects, and interactions between county and stack fixed effects. Adjusted models additionally control for patient age, patient race, and the Elixhauser Comorbidity Index. We estimated robust standard errors clustered by patient’s county of residence. Obstetric trauma for admissions with an instrument is an Agency for Healthcare Research and Quality (AHRQ) Patient Safety Indicator (PSI 18) measuring severe perineal lacerations during instrument-assisted vaginal deliveries. The sample size for this outcome is 18 104. Obstetric trauma for admissions without use of an instrument is an AHRQ Patient Safety Indicator (PSI 19) measuring severe perineal lacerations during spontaneous vaginal deliveries without the use of instruments.

Prior to a merger, 37.6% of admissions occurred in safety net hospitals in counties later experiencing a merger. Mergers were associated with an adjusted 9.2–percentage point (95% CI, 2.3-16.1) increase in the probability of admission to a safety net hospital. At baseline, 72.2% of Medicaid admissions occurred at a hospital with an NICU. Mergers were associated with a 7.9–percentage point (95% CI, −11.2 to −4.6) adjusted decrease in probability of admission to an NICU-equipped hospital. In secondary analyses, we found that mergers were associated with a 4.7–percentage point (95% CI, –6.7 to –1.3) reduction in the share of hospitals with an NICU in a patient’s county (eTable 5 in Supplement 1).

Mergers were not associated with changes in rates of obstetric trauma for admissions with instrument (adjusted difference-in-differences estimate: 1.6; 95% CI, −0.8 to 4.0). We observed a small increase in obstetric trauma rates for admissions without instrument (adjusted difference-in-differences estimate: 0.4; 95% CI, 0.2-0.6). Only 0.1% of admissions experienced in-hospital mortality at baseline, and mergers were not associated with changes in these rates.

We did not observe evidence of differential premerger trends in outcomes between admissions in merger vs nonmerger counties (eFigure 1 in Supplement 1), supporting the underlying assumptions of the model. Moreover, we observed a modest increase in the share of Hispanic patient admissions, but we did not observe changes in any other patient characteristics following mergers, suggesting that mergers did not substantially alter the composition of Medicaid enrollees served at a given hospital (eTable 6 in Supplement 1).

### Urban/Rural Stratification

Mergers were associated with a greater increase in distance traveled in rural counties (22.8 miles, 95% CI, 19.4-26.2) vs urban counties (0.6 miles, 95% CI, 0.1-1.0) (Table 3). Mergers were associated with a 7.7–percentage point (95% CI, 1.4-14.0) increase in likelihood of admission to a safety net hospital in rural counties and an 11.7–percentage point (95% CI, 4.3-19.1) increase in urban counties. In rural counties, mergers were associated with a large increase in probability of admission to an NICU-equipped hospital (79.8, 95% CI, 71.8-87.8), compared with a 10–percentage point (95% CI, −13.5 to −6.5) decrease in urban counties. Mergers were not associated with estimated changes in quality outcomes in rural markets. In urban markets, a hospital merger was associated with a 0.3–percentage point (95% CI, 0.1-0.5) increase in obstetric trauma rates among admissions without an instrument, but not with changes in other outcomes. Although event-study analyses did not detect differential outcome trends in premerger years, estimates in rural markets were imprecise due to small sample sizes (eFigure 2 in Supplement 1).

**Table 3.** Urban vs Rural Stratification: Stacked Difference-in-Differences Estimates of the Association Between County-Level Exposure to a Hospital Merger and Hospital Choice and Outcomes For Medicaid Labor and Delivery Hospital Admissions^a^

Outcome	Premerger outcomes in counties with a hospital merger, %	Stacked difference-in-differences estimate (95% CI)	
		Unadjusted	Adjusted
**Number of Medicaid admissions in rural counties (n = 5827)**			
Patient access and hospital choice			
Patient distance to hospital, miles	17.9	23.2 (20.2 to 26.1)	22.8 (19.4 to 26.2)
Admission to safety net hospital	6.9	8 (2.1 to 13.9)	7.7 (1.4 to 14.0)
Admission to hospital with an NICU	34.9	81.3 (73.5 to 89.1)	79.8 (71.8 to 87.8)
Obstetric quality outcomes			
Obstetric trauma, instrument-assisted deliveries	5.1	−1.6 (−16.3 to 13.1)	7.5 (−8.4 to 23.4)
Obstetric trauma, non-instrument-assisted deliveries	1.0	−1.6 (−3.8 to 0.6)	−1.5 (−3.7 to 0.7)
In-hospital death	0	0 (0 to 0)	0 (0 to 0)
**Number of Medicaid admissions in urban counties (n = 480 377)**			
Patient access and hospital choice			
Patient distance to hospital, miles	5.5	0.5 (0.1 to 1)	0.6 (0.1 to 1)
Admission to safety net hospital	36.8	8.2 (0.8 to 15.6)	11.7 (4.3 to 19.1)
Admission to hospital with an NICU	78.2	−10.4 (−14.1 to −6.7)	−10 (−13.5 to −6.5)
Obstetric quality outcomes			
Obstetric trauma, instrument-assisted deliveries	14.3	1.4 (−1.1 to 3.9)	1.8 (−0.7 to 4.3)
Obstetric trauma, non–instrument-assisted deliveries	2.0	0.2 (0 to 0.4)	0.3 (0.1 to 0.5)
In-hospital death	0.01	0 (0 to 0)	0 (0 to 0)

Abbreviation: NICU, neonatal intensive care unit.

^a^ Analyses are stratified by admissions in counties designated as rural or urban. Urban refers to core-based statistical areas designated as Metropolitan. Rural refers to counties outside core-based statistical areas. Stacked difference-in-differences coefficient estimates and 95% CIs from the main analysis are presented as well as mean outcomes across admissions in merger counties during premerger years. Model specifications and estimation are the same as that in the main analysis.

### Differential Impacts for Privately Insured vs Medicaid

We found differential effects of mergers for patients with Medicaid compared with the privately insured for certain outcomes (Table 4). In contrast to the effects seen among patients with Medicaid, mergers did not result in an increase in distance traveled for privately insured patients. Privately insured patients also did not experience increases in admission to a safety-net hospital after a merger (1 percentage point, 95% CI, −1.6 to 3.6). Both privately insured and Medicaid patients experienced similar decreases in likelihood of admission to a hospital with an NICU. Differences in quality outcomes for privately insured and Medicaid patients were not statistically significant.

**Table 4.** Associations Between County-Level Exposure to Hospital Mergers and Outcomes Among Privately Insured Patients Admitted for Labor and Delivery, and Differential Associations for Medicaid Enrollees^a^

Outcome	Association between hospital merger in county and outcomes		
	Privately insured patients, adjusted, estimate (95% CI)	Medicaid patients relative to association for privately insured, differential, estimate (95% CI)	*P* value
Total admissions, N = 1 330 199			
Patient access and hospital choice			
Patient distance to hospital, miles	0 (−0.3 to 0.3)	0.6 (0 to 1.1)	.05
Admission to safety net hospital	1 (−1.6 to 3.6)	6.8 (1 to 12.6)	.02
Admission to hospital with a neonatal intensive care unit	−7.6 (−10.2 to −5.1)	−0.1 (−2.4 to 2.3)	.97
Obstetric quality outcomes			
Obstetric trauma, instrument-assisted deliveries	−0.7 (−2.6 to 1.2)	2.3 (−0.7 to 5.3)	.13
Obstetric trauma, non–instrument-assisted deliveries	0.1 (−0.1 to 0.4)	0.3 (−0.1 to 0.6)	.12
In-hospital death	0 (0 to 0)	0 (0 to 0)	.84

^a^ This analysis includes admissions for labor and delivery admissions for both privately insured and Medicaid enrollees in counties with a merger and matched comparison counties with no merger. Stacked difference-in-differences framework was modified to estimate the association between county-exposure to a merger and outcomes for private insurance admissions and differential effects in this association for Medicaid admissions. We modified our main model to include interactions between patient insurance status with treatment group and postmerger indicators, stack fixed effects, stack-year fixed effects, and stack-county fixed effects in addition to the terms in the main model. Other aspects of the model estimation approach are consistent with the main analyses.

## Discussion

This study found that hospital mergers were associated with several changes in where patients with Medicaid received labor and delivery care and with modest declines in 1 obstetric quality outcome. Specifically, among Medicaid enrollees, hospital mergers were associated with increased travel distances for labor and delivery admissions, greater reliance on safety net hospitals, and reduced use of NICU-equipped hospitals. Merger-related changes in admission patterns among Medicaid enrollees varied by market context, with labor and delivery admissions to NICU-equipped hospitals increasing in rural markets but declining in urban markets. In addition, mergers were associated with larger increases in both distance and safety net hospital admission among Medicaid enrollees than among privately insured patients. Although we find an association between mergers and obstetric trauma involving non–instrument-assisted deliveries among Medicaid enrollees, other obstetric quality outcomes did not change significantly among either Medicaid or commercially insured patients. These findings suggest that hospital mergers may be associated with negative changes in access to care, and perhaps quality, for publicly insured patients, while highlighting salient differences in these outcomes by market context.

This cross-sectional study provides new evidence that hospital mergers are associated with distinct changes in care received by Medicaid enrollees, a population often underrepresented in research on hospital consolidation. It suggests that mergers are associated with changes in how hospital care is distributed among vulnerable groups.^31,32,33^ The larger increases in travel distance and safety net hospital use we observe for those with Medicaid relative to privately insured patients may stem from more limited care networks and fewer options for patients with Medicaid, which make them more responsive than privately insured patients to merger-associated changes in hospital access and referral patterns. These results underscore the importance of considering low-income populations when evaluating the full range of merger consequences.

These findings also offer new insights into how hospital mergers, which are related to but distinct from closures, may affect labor and delivery care in rural markets. Consistent with the closures literature, consolidation can force patients to travel greater distances for essential care. However, also consistent with previous literature,^34,35^ we found that mergers in rural markets were associated with an increase in admissions to hospitals with NICUs, which may indicate that patients are receiving specialized care in better-equipped hospitals. However, we do not detect changes in quality outcomes in rural markets, potentially due to the varied ways in which mergers influence care and the limited statistical power to detect relatively rare outcomes in this subsample.

In urban markets, we observed a decline in admissions to hospitals with NICU facilities and a small increase in obstetric trauma rates for admissions without use of an instrument, suggesting that, although overall access concerns may be less pronounced in urban areas, patients might be funneled toward lower-resourced hospitals. Finally, in both urban and rural markets, mergers were associated with increased admissions at safety-net hospitals among patients with Medicaid, underscoring the critical role these facilities play.

### Limitations

This longitudinal study had several limitations. First, the analysis focused on years that mostly predate Medicaid expansion included in the Affordable Care Act (ACA), which states began to implement in 2014. The ACA expansion broadened Medicaid eligibility for adults who did not qualify for Medicaid based on pregnancy, old age, or a disability. Although we do not expect eligibility expansions to substantially alter the composition of our population because they did not apply to pregnant populations, prior evidence of Medicaid woodwork effects suggests that expansion could have affected Medicaid enrollment for some already-eligible individuals.^36,37,38,39^ This could increase coverage continuity or shift hospital payer mix, potentially influencing care seeking patterns or hospital responses to consolidation. Future research could assess whether such dynamics modify merger impacts in the postexpansion period.

Second, our analysis focused on 9 states. Although these states represent approximately 25% of US births, our estimates may not fully capture the effects of hospital mergers in other regions. Third, hospital mergers are not randomly assigned. Although we implemented matching on premerger trends, conducted event-study analyses to test the parallel trends assumption, and found no meaningful differences in most observable patient characteristics at baseline, unobserved time-varying factors (eg, changes in Medicaid enrollment practices across markets) could introduce bias. We did observe a modest increase in the share of Hispanic patients associated with mergers, highlighting the potential for small compositional shifts that may not be fully captured in our data. Fourth, the smaller sample sizes in rural areas make these estimates more sensitive to random fluctuations; consequently, these findings should be interpreted with caution. Fifth, our patient travel distance measure may introduce measurement error, as actual travel routes may differ from the straight-line calculation we used. Finally, although we examined key quality measures such as obstetric trauma and maternal mortality, our data did not allow us to capture other indicators of maternal care quality, such as delivery type (eg, vaginal birth, unnecessary cesarean delivery) or postpartum outcomes.

## Conclusions

This cross-sectional study of labor and delivery admissions among patients with Medicaid found that hospital mergers were associated with an increased probability of admission to safety-net hospitals, lower probability of NICU-equipped hospitals, and worsening in 1 obstetric quality metric. These changes differed for Medicaid vs commercially insured patients and varied among Medicaid patients in urban vs rural markets. As hospital markets continue to consolidate, it is imperative for policymakers, regulators, and Medicaid agencies to recognize potential risks to low-income populations.

## Notes

Supplement 1. eMethods eReferences eTable 1. County-Level Characteristics Used in the Matching Model: Merger vs. Matched Comparison Counties eTable 2. Pre-matching descriptive statistics: Medicaid labor and delivery admissions in merger and all non-merger counties (prior to comparison group selection through matching) eTable 3. Counties excluded due to multiple mergers during the study period: Descriptive characteristics of Medicaid labor and delivery admissions in counties experiencing multiple mergers. eTable 4. Privately insured patient population: Descriptive characteristics of privately insured labor and delivery admissions in counties with hospital mergers and matched comparison counties eTable 5. Difference-in-differences estimate of the association between county-level exposure to a hospital merger and availability of an NICU in the county eTable 6. Stacked difference-in-differences estimates of the association between county-level exposure to a hospital merger and hospital choice and admitted Medicaid patient characteristics eFigure 1. Event-study estimates of the association between a county-level exposure to a hospital merger and outcomes for Medicaid enrollees admitted to a hospital for labor and delivery eFigure 2. Urban/rural stratification: Event-study estimates of the association between a county-level exposure to a hospital merger and hospital choice outcomes for Medicaid enrollees admitted to a hospital for labor and delivery

Supplement 2. Data Sharing Statement
