# Supplementary material for: Hospital Mergers, Hospital Choice, and Care Quality for Pregnant Enrollees in Medicaid
Source: JAMA Health Forum. 2025 Dec 5;6(12):e255334. doi: 10.1001/jamahealthforum.2025.5334 (PMC12681039; doi:10.1001/jamahealthforum.2025.5334)
Supplement: Supplement 1. — eMethods eReferences eTable 1. County-Level Characteristics Used in the Matching Model: Merger vs. Matched Comparison Counties eTable 2. Pre-matching descriptive statistics: Medicaid labor and delivery admissions in merger and all non-merger counties (prior to comparison group selection through matching) eTable 3. Counties excluded due to multiple mergers during the study period: Descriptive characteristics of Medicaid labor and delivery admissions in counties experiencing multiple mergers. eTable 4. Privately insured patient population: Descriptive characteristics of privately insured labor and delivery admissions in counties with hospital mergers and matched comparison counties eTable 5. Difference-in-differences estimate of the association between county-level exposure to a hospital merger and availability of an NICU in the county eTable 6. Stacked difference-in-differences estimates of the association between county-level exposure to a hospital merger and hospital choice and admitted Medicaid patient characteristics eFigure 1. Event-study estimates of the association between a county-level exposure to a hospital merger and outcomes for Medicaid enrollees admitted to a hospital for labor and delivery eFigure 2. Urban/rural stratification: Event-study estimates of the association between a county-level exposure to a hospital merger and hospital choice outcomes for Medicaid enrollees admitted to a hospital for labor and delivery [file jamahealthforum-e255334-s001.pdf]

Supplemental Online Content

Desai SM, Padmanabhan P, Glied S, Roberts ET. Hospital mergers, hospital choice, and care quality for pregnant enrollees in Medicaid. *JAMA Health Forum.* 2025;6(12):e255334. doi:10.1001/jamahealthforum.2025.5334

eMethods
eReferences
eTable 1. County-Level Characteristics Used in the Matching Model: Merger vs. Matched Comparison Counties
eTable 2. Pre-matching descriptive statistics: Medicaid labor and delivery admissions in merger and all non-merger counties (prior to comparison group selection through matching)
eTable 3. Counties excluded due to multiple mergers during the study period: Descriptive characteristics of Medicaid labor and delivery admissions in counties experiencing multiple mergers
eTable 4. Privately insured patient population: Descriptive characteristics of privately insured labor and delivery admissions in counties with hospital mergers and matched comparison counties
eTable 5. Difference-in-differences estimate of the association between county-level exposure to a hospital merger and availability of a NICU in the county
eTable 6. Stacked difference-in-differences estimates of the association between county-level exposure to a hospital merger and hospital choice and admitted Medicaid patient characteristics
eFigure 1. Event-study estimates of the association between a county-level exposure to a hospital merger and outcomes for Medicaid enrollees admitted to a hospital for labor and delivery
eFigure 2. Urban/rural stratification: Event-study estimates of the association between a county-level exposure to a hospital merger and hospital choice outcomes for Medicaid enrollees admitted to a hospital for labor and delivery

This supplemental material has been provided by the authors to give readers additional information about their work.

© 2025 Desai SM et al. *JAMA Health Forum.*

A. METHODOLOGICAL DETAILS

1. Comparison county selection: Propensity score matching

We used propensity score matching to identify comparison counties. We estimated propensity score models using logistic regression and controlling for baseline year (3 years before the merger or concurrent year) county-level characteristics: total number hospitals in the county, total number of hospital beds in the county, baseline HHI level, county-level uninsured rate, trends for the proportion of admissions to a safety net hospital (measured as the year-to-year change in this proportion during pre-merger years up until the merger year), trends for the proportion of admissions to a hospital with a NICU, and trends in Herfindahl-Hirschmann Index (HHI). HHI measures the concentration of hospital admissions within a market and is scaled from 0 to 10,000, with higher values indicating greater concentration. We required an exact match based on the county's core-based statistical area designation (CBSA) of metropolitan, micropolitan, or outside CBSAs. Each intervention county was matched to one comparison county using nearest neighbor matching based on a predicted propensity score. Matching was done by stack (year of merger), and matching was conducted without replacement within a stack but with replacement across stacks.

2. Herfindahl-Hirschmann Index (HHI) Construction

We constructed an zip code-specific HHI measure adapted from the approaches of Capps et. al. (2017), Desai et. al. (2021), and Kessler and McClellan (2000).[1–3] Our HHI measure essentially reflects the market concentration (HHIs) in markets served by hospitals in a patient's county (as opposed to only those hospitals located in a the county). The advantage of this HHI relative to an HHI using a fixed geography is that it reflects the market environment across all the markets a

© 2025 Desai SM et al. *JAMA Health Forum*.

hospital services and in turn responds to, rather than assuming that each hospital only operates within arbitrary geographic boundaries.

Our HHI measure was constructed using labor and delivery admissions for privately insured patients. We used admissions for privately-insured patients to reflect the hypothesized mechanism underlying our conceptual framework that changes in market power and prices in privately insured markets could impact care for low-income, publicly-insured patients.

We construct the HHI in four steps. First, we calculate a standard HHI for each county in each year, defining market shares at the hospital system-level. (Hospital system affiliations for each hospital were obtained from the American Hospital Association Annual Survey database.) This county HHI is given by the sum of the squared market shares for all hospital systems with inpatient admissions for patients in the given county. Specifically, the numerator for each hospital system's market share is the number of admissions across all hospitals affiliated with a given hospital system in that year. The denominator for each hospital's market share is the total number of admissions for all patient's residing in that particular county. Within each county, we square these hospital-system market shares and sum them.

Second, we calculate the weighted average of these county HHIs for each hospital, where the weight applied to each county HHI is given by the proportion of that hospital's admissions that are for patients residing in the county.

Third, we calculate the weighted average of these hospital HHIs for each county, where the weight applied to the HHI of each hospital that services a county is given by the proportion of that hospital's market share in that county.

Finally, county-specific HHI measures were scaled from 0 to 10,000.

3. Model specifications

a. Main model specification

© 2025 Desai SM et al. *JAMA Health Forum*.

In our stacked difference-in-differences, we specify the following model:

$$y_{icts} = \beta_0 + \beta \text{Post}_{is} * \text{Treat}_{sc} + \gamma \mathbf{X}_{icts} + \boldsymbol{\alpha}_s + \boldsymbol{\alpha}_s * \boldsymbol{\alpha}_c + \boldsymbol{\alpha}_s * \boldsymbol{\alpha}_t + \varepsilon_{icts}$$

where y_{icts} denotes the outcome for admission *i* for patient residing in county *c* in stack s in year *t*, Post_{cts} denotes whether the admission occurred in the stack s's post-merger period, $Treat_{isc}$ is whether county c was a merger county, X_{icts} is a vector of admission characteristics (patient age, race, and Elixhauser comorbidity index), α_s are stack fixed effects, $\alpha_s * \alpha_c$ denote stack-county fixed effects, $\alpha_s * \alpha_t$ denote stack-year fixed effects, and ε_{icts} is a random error term.

b. Event study model

In our event study specification of the stacked difference-in-differences, we modify the model to estimate year-specific difference-in-differences estimates for each year relative to the year before the merger. Specifically, we specify the following model:

$$y_{icts} = \beta_0$$
$$+ \beta_1 \mathbf{1(year{=}merger{-}3)}_{is} * \text{Treat}_{sc} + \beta_2 \mathbf{1\ (year{=}merger{-}2)}_{is} * \text{Treat}_{sc}$$
$$+ \beta_3 \mathbf{1\ (year{=}merger{+}1)}_{is} * \text{Treat}_{sc} + \beta_4 \mathbf{1\ (year{=}merger{+}2)}_{is} * \text{Treat}_{sc} + \beta_5 \mathbf{1\ (year{=}merger{+}3)}_{is} * \text{Treat}_{sc}$$
$$+ \gamma \mathbf{X}_{icts} + \alpha_s + \alpha_s * \alpha_c + \alpha_s * \alpha_t + \varepsilon_{icts}$$

where **1 (year=merger-3)** is an indicator function denoting whether admission i occurred 3 years prior to the merger year, **1 (year=merger-2)** denotes whether admission i occurred 2 years prior to the merger year, **1 (year=merger+1)** denotes whether admission i occurred 1 years after the merger year, **1 (year=merger+2)** denotes whether admission i occurred 2 years after the merger year, and **1 (year=merger+3)** denotes whether admission i occurred 3 years after the merger year.

© 2025 Desai SM et al. *JAMA Health Forum*.

c. Interaction model to test for differential effects for privately insured versus Medicaid enrollee admissions

We modified our stacked difference-in-differences to estimate differential effects in outcomes for Medicaid enrollees compared to privately insured enrollees. The modified model specification is given by

$$y_{icts} = \beta_0 + \beta_1 Post_{is} * Treat_{sc} + \beta_2 Post_{is} * Treat_{sc} * Medicaid_{isct}\ \gamma \mathbf{X_{icts}} + \alpha_s + \alpha_s * \alpha_c + \alpha_s * \alpha_t + \alpha_s * \alpha_m + \alpha_s * \alpha_c * \alpha_m + \alpha_s * \alpha_t * \alpha_m\ \varepsilon_{icts}$$

where $Medicaid_{isct}$ is an indicator for whether admission i is for a Medicaid enrollee (vs. a privately insured patient) and α_m denote fixed effect indicator variables for whether the admission is for a Medicaid enrollee as well. In this model, β_1 reflects the difference-in-differences coefficient for privately insured patients and β_2 reflects the differential effect for Medicaid patients relative to that for privately insured patients.

© 2025 Desai SM et al. *JAMA Health Forum*.

B. REFERENCES

1. Capps C, Dranove D, Ody C. The effect of hospital acquisitions of physician practices on prices and spending. *Journal of Health Economics*. 2018;59:139-152. doi:https://doi.org/10.1016/j.jhealeco.2018.04.001

2. Desai SM, Padmanabhan P, Chen AZ, Lewis A, Glied SA. Hospital concentration and low-income populations: Evidence from New York State Medicaid. *Journal of Health Economics*. 2023;90:102770. doi:10.1016/j.jhealeco.2023.102770

3. Kessler DP, McClellan MB. Is Hospital Competition Socially Wasteful?. *The Quarterly Journal of Economics*. 2000;115(2):577-615. doi:10.1162/003355300554863

© 2025 Desai SM et al. *JAMA Health Forum*.

C. Tables and Figures

Table S1. County-Level Characteristics Used in the Matching Model: Merger vs. Matched Comparison Counties

	Counties with a hospital merger		Matched counties with no hospital merger in concurrent time period	
	Pre-merger	Post-merger	Pre-merger	Post-merger
**County-year level characteristics**[c]				
No. hospitals in county	15.8	16.6	15.0	14.7
No. hospital beds in county	6283.0	6528.0	5854.0	6043.0
Uninsured Rate, %	6.9	4.0	5.6	4.7
HHI (Mean, SD)[d]	4376 (1305)	5703 (1362)	4367 (1330)	4489 (1282)
Trends (year-to-year change):				
HHI, % change	2.3	1.9	2.4	-0.1
Proportion admitted to safety net hospital, pp	1.4	0.5	0.7	0.9
Proportion admitted to hospital with NICU, pp	2.9	0.1	3.2	1.3

© 2025 Desai SM et al. *JAMA Health Forum*.

Table S2. Pre-matching descriptive statistics: Medicaid labor and delivery admissions in merger and all non-merger counties (prior to comparison group selection through matching)

	Counties with a hospital merger		All counties with no hospital merger in concurrent time period	
	Pre-merger	Post-merger	Pre-merger	Post-merger
No. unique counties	30	30	286	286
No. admissions	159,580	159,007	3,686,391	3,946,001
Age (Mean, SD)	25.4 (5.9)	26.1 (5.9)	24.6 (6.5)	25.2 (5.7)
Race and ethnicity, No. (%)				
White	32,739 (20.5)	48,752 (30.7)	1,423,440 (38.6)	1,643,331 (41.6)
Black	24,629 (15.4)	22,984 (14.5)	667,058 (18.1)	799,268 (20.3)
Hispanic	31,713 (19.9)	52,178 (32.8)	633,404 (17.2)	818,480 (20.7)
Other	70,499 (44.2)	35,093 (22.1)	962,489 (26.1)	684,922 (17.4)
Elixhauser Index, No. %				
0	137,713 (86.3)	130,481 (82.1)	3,135,107 (85)	3,201,004 (81.1)
1	18,396 (11.5)	23,466 (14.8)	460,512 (12.5)	611,342 (15.5)
2	2,873 (1.8)	4,242 (2.7)	766,12 (2.1)	112,898 (2.9)
3+	598 (0.4)	818 (0.5)	14,160 (0.3)	20,757 (0.6)
HHI - County (Mean, SD)[c]	3,264 (1385)	3,638 (1311)	4,712 (1589)	4,706 (1518)
Core-Based Statistical Area (CBSA) Category, No. (%)				
Metropolitan (Urban)	144,465 (90.5)	142,467 (89.6)	2,932,735 (79.6)	3,202,411 (81.2)
Micropolitan	11,658 (7.3)	13,762 (8.7)	492,685 (13.4)	493,079 (12.5)
Outside CBSA (Rural)	3,457 (2.2)	2,778 (1.8)	260,971 (7.1)	250,511 (6.3)

© 2025 Desai SM et al. *JAMA Health Forum*.

Table S3. Counties excluded due to multiple mergers during the study period: Descriptive characteristics of Medicaid labor and delivery admissions in counties experiencing multiple mergers.

	Counties with multiple hospital mergers	
	Pre-merger	Post-merger
No. unique counties	30	30
No. admissions	501,289	482,239
Age (Mean, SD)	25.7 (6.1)	26.4 (6.1)
Race and ethnicity, No. (%)		
White	116,158 (23.2)	133,886 (27.8)
Black	106,488 (21.2)	97,486 (20.2)
Hispanic	185,114 (36.9)	172,554 (35.8)
Other	93,529 (18.7)	78,313 (16.2)
Elixhauser Index, No. %		
0	137713 (86.3)	130481 (82.1)
1	18396 (11.5)	23466 (14.8)
2	2873 (1.8)	4242 (2.7)
3+	598 (0.4)	818 (0.4)
HHI (Mean, SD)[c]	3,013 (1,144)	3,201 (1,196)
Core-Based Statistical Area (CBSA) Category, No. (%)		
Metropolitan (Urban)	497,401 (99.2)	478,588 (99.2)
Micropolitan	3,263 (0.7)	3,027 (0.6)
Outside CBSA (Rural)	625 (0.1)	622 (0.1)

Note: Pre-merger is defined as the three years prior to the first merger in the system, and post-merger is the three years following the last merger in the county. These counties experienced 2.53 merger events on average.

© 2025 Desai SM et al. *JAMA Health Forum*.

Table S4. Privately insured patient population: Descriptive characteristics of privately insured labor and delivery admissions in counties with hospital mergers and matched comparison counties

	Counties with a hospital merger		Matched counties with no hospital merger in concurrent time period	
	Pre-merger	Post-merger	Pre-merger	Post-merger
No. unique counties	30	30	28	28
No. admissions	206,226	185,689	213478	197315
Age (Mean, SD)	30.5 (5.7)	30.6 (5.5)	31.2 (5.5)	31.4 (5.4)
Race and ethnicity, No. (%)				
White	108,052 (52.4)	115,751 (62.3)	149,213 (69.9)	142,670 (72.3)
Black	14,376 (7.0)	13,089 (7.0)	12,754 (6.0)	11,620 (5.9)
Hispanic	13,622 (6.6)	15,325 (8.3)	14,370 (6.7)	13,324 (6.8)
Other	70,176 (34.0)	41,524 (22.4)	37,141 (17.4)	29,701 (15.1)
Elixhauser Index, No. %				
0	180626 (87.6)	156893 (84.5)	187093 (87.6)	167407 (84.8)
1	22551 (10.9)	24987 (13.5)	23356 (10.9)	26154 (13.3)
2	2665 (1.3)	3372 (1.8)	2677 (1.3)	3375 (1.7)
3+	384 (0.2)	437 (0.2)	352 (0.1)	379 (0.2)
HHI (Mean, SD)[c]	3405 (1143)	3703 (1180)	3199 (1268)	3233 (1266)
Core-Based Statistical Area (CBSA) Category, No. (%)				
Metropolitan (Urban)	192006 (93.1)	174099 (93.8)	205101 (96.1)	188780 (95.7)
Micropolitan	11616 (5.6)	8743 (4.7)	7560 (3.5)	7814 (4.0)
Outside CBSA (Rural)	2604 (1.3)	2847 (1.5)	817 (0.4)	721 (0.4)

© 2025 Desai SM et al. *JAMA Health Forum*.

Table S5. Difference-in-differences estimate of the association between county-level exposure to a hospital merger and availability of a NICU in the county

No. admissions: 527,499	Pre-merger outcomes in counties with a hospital merger	Unadjusted stacked difference-in-differences estimate (95% CI)	Adjusted stacked difference-in-differences estimate (95% CI)
Availability of a NICU in patient's county	78.9	-4.7 (-6.7, -1.3)	-4.7 (-6.7, -1.3)

© 2025 Desai SM et al. *JAMA Health Forum*.

Table S6. Stacked difference-in-differences estimates of the association between county-level exposure to a hospital merger and hospital choice and admitted Medicaid patient characteristics

Outcome	Counties with a hospital merger		Matched counties with no hospital merger in concurrent time period		Unadjusted difference-in-differences estimate (95% CI)
	Pre-merger	Post-merger	Pre-merger	Post-merger	
Median Household Income, $	55058	57077	68952	69964	0 (0, 0)
Age	25.5	26.1	25.7	26.3	0.083 (0, 0.17)
Race, %					
White	0.295	0.362	0.368	0.373	0.033 (0.01, 0.06)
Black	0.208	0.171	0.194	0.187	-0.005 (-0.02, 0.01)
Hispanic	0.291	0.387	0.309	0.277	0.133 (0.1, 0.16)
SMM	0.018	0.024	0.022	0.026	0.001 (0, 0)
Elixhauser					
0	0.819	0.792	0.809	0.777	0.006 (0, 0.01)
1	0.120	0.148	0.122	0.156	-0.004 (-0.01, 0)
2	0.019	0.027	0.021	0.032	-0.002 (0, 0)
3	0.003	0.004	0.004	0.005	-0.001 (0, 0)
4	0.001	0.011	0.001	0.001	0 (0, 0)
5	0.009	0.008	0.007	0.007	0 (0, 0)
6	0.000	0.004	0.000	0.001	0 (0, 0)

© 2025 Desai SM et al. *JAMA Health Forum*.

Figure S1. Event-study estimates of the association between a county-level exposure to a hospital merger and outcomes for Medicaid enrollees admitted to a hospital for labor and delivery

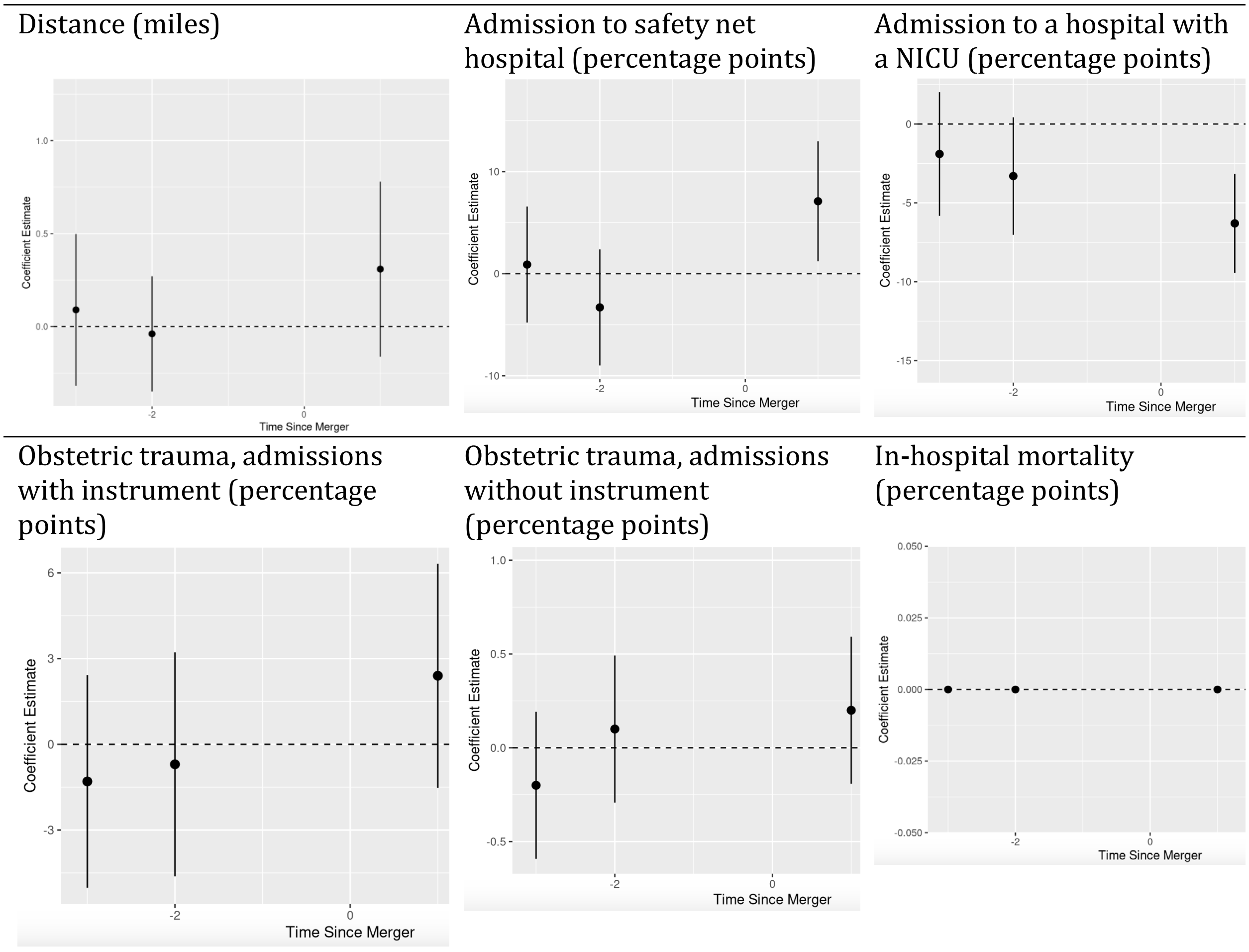

Note: This figure presents event-study estimates from the adjusted stacked difference-in-differences framework, where the outcome variable is the average distance (in miles) traveled by patients. The coefficients plotted correspond to the effect of a merger in the two years prior to the merger and the three years following it, all relative to the year immediately preceding the merger (the omitted reference period). Each point on the plot is the estimated effect for that relative year, with vertical lines indicating the 95% confidence intervals. A positive (negative) coefficient reflects an increase (decrease) in how far patients travel after the merger, compared to the baseline year.

© 2025 Desai SM et al. *JAMA Health Forum.*

Figure S2. Urban/rural stratification: Event-study estimates of the association between a county-level exposure to a hospital merger and hospital choice outcomes for Medicaid enrollees admitted to a hospital for labor and delivery

	Distance (miles)	Admission to safety net hospital (percentage points)	Admission to a hospital with a NICU (percentage points)
Rural county admissions (Outside CBSAs)	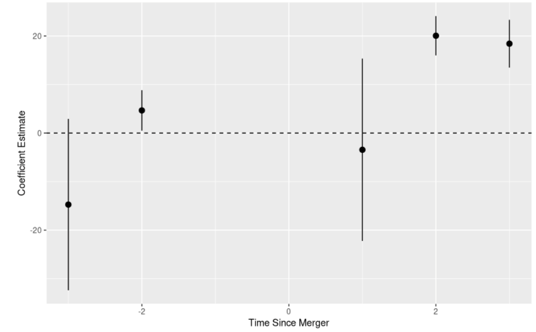	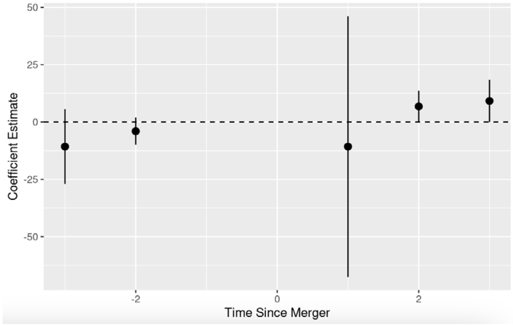	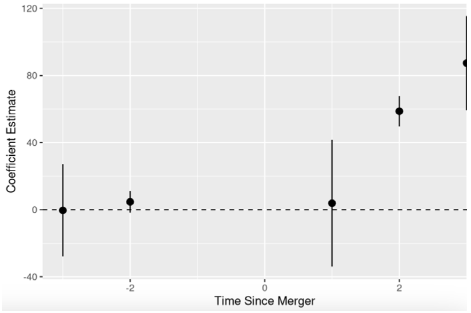
Urban county admissions (Metropolitan CBSAs)	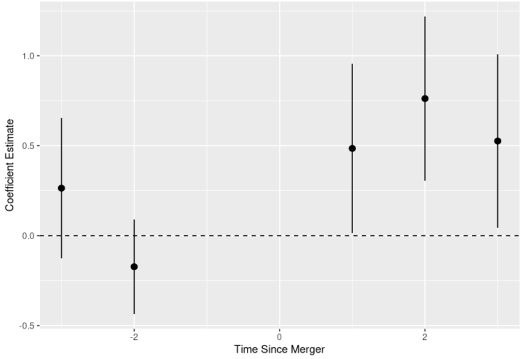	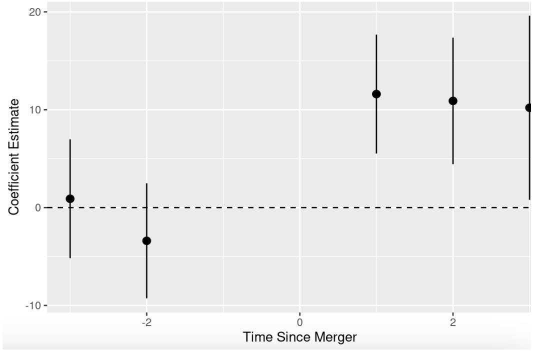	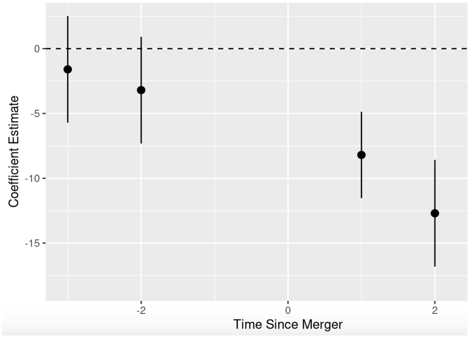

© 2025 Desai SM et al. *JAMA Health Forum*.
