# Supplementary material for: Hospital Mergers, Hospital Choice, and Care Quality for Pregnant Enrollees in Medicaid
Source: JAMA Health Forum. 2025 Dec 5;6(12):e255334. doi: 10.1001/jamahealthforum.2025.5334 (PMC12681039; doi:10.1001/jamahealthforum.2025.5334)
Supplement: Supplement 2. — Data Sharing Statement [file jamahealthforum-e255334-s002.pdf]

Data Sharing Statement

Desai. Hospital Mergers, Hospital Choice, and Care Quality for Pregnant Enrollees in Medicaid. *JAMA Health Forum*. Published December 05, 2025.
doi:10.1001/jamahealthforum.2025.5334

Data
Data available: Yes
Data types: Data dictionary
How to access data: Researchers seeking data dictionary and code can contact Sunita Desai at sunita.desai@nyu.edu
When available: With publication

Supporting Documents
Document types: Statistical/analytic code
How to access documents: Researchers seeking data dictionary and code can contact Sunita Desai at sunita.desai@nyu.edu
When available: With publication

Additional Information
Who can access the data: researchers whose proposed use of the data has been approved
Types of analyses: Research purposes
Mechanisms of data availability: With approval
